# Pursuing equity in cancer care: implementation, challenges and preliminary findings of a public cancer referral center in rural Rwanda

**DOI:** 10.1186/s12885-016-2256-7

**Published:** 2016-03-18

**Authors:** Neo M. Tapela, Tharcisse Mpunga, Bethany Hedt-Gauthier, Molly Moore, Egide Mpanumusingo, Mary Jue Xu, Ignace Nzayisenga, Vedaste Hategekimana, Denis Gilbert Umuhizi, Lydia E. Pace, Jean Bosco Bigirimana, JingJing Wang, Caitlin Driscoll, Frank R. Uwizeye, Peter C. Drobac, Gedeon Ngoga, Cyprien Shyirambere, Clemence Muhayimana, Leslie Lehmann, Lawrence N. Shulman

**Affiliations:** Botswana Ministry of Health, Gaborone, Botswana; Partners In Health/Inshuti Mu Buzima, Kigali, Rwanda; Dana-Farber/Brigham & Women’s Cancer Center, Boston, USA; Harvard Medical School, Boston, USA; Rwanda Ministry of Health, Kigali, Rwanda; Boston Children’s Hospital, Boston, USA; University of Vermont College of Medicine, Burlington, USA; Icahn School of Medicine at Mount Sinai, New York, USA; Abramson Cancer Center, University of Pennsylvania, Philadelphia, USA; Division of Global Health Equity, Brigham and Women’s Hospital, Boston, USA

**Keywords:** Cancer, Implementation, Rwanda, Resource-limited setting, Capacity building, Twinning, Task-shifting

## Abstract

**Background:**

Cancer services are inaccessible in many low-income countries, and few published examples describe oncology programs within the public sector. In 2011, the Rwanda Ministry of Health (RMOH) established Butaro Cancer Center of Excellence (BCCOE) to expand cancer services nationally. In hopes of informing cancer care delivery in similar settings, we describe program-level experience implementing BCCOE, patient characteristics, and challenges encountered.

**Methods:**

Butaro Cancer Center of Excellence was founded on diverse partnerships that emphasize capacity building. Services available include pathology-based diagnosis, basic imaging, chemotherapy, surgery, referral for radiotherapy, palliative care and socioeconomic access supports. Retrospective review of electronic medical records (EMR) of patients enrolled between July 1, 2012 and June 30, 2014 was conducted, supplemented by manual review of paper charts and programmatic records.

**Results:**

In the program’s first 2 years, 2326 patients presented for cancer-related care. Of these, 70.5 % were female, 4.3 % children, and 74.3 % on public health insurance. In the first year, 66.3 % (*n* = 1144) were diagnosed with cancer. Leading adult diagnoses were breast, cervical, and skin cancer. Among children, nephroblastoma, acute lymphoblastic leukemia, and Hodgkin lymphoma were predominant. As of June 30, 2013, 95 cancer patients had died. Challenges encountered include documentation gaps and staff shortages.

**Conclusion:**

Butaro Cancer Center of Excellence demonstrates that complex cancer care can be delivered in the most resource-constrained settings, accessible to vulnerable patients. Key attributes that have made BCCOE possible are: meaningful North–south partnerships, innovative task- and infrastructure-shifting, RMOH leadership, and an equity-driven agenda. Going forward, we will apply our experiences and lessons learned to further strengthen BCCOE, and employ the developed EMR system as a valuable platform to assess long-term clinical outcomes and improve care.

## Background

As cancer-related mortality rapidly outpaces the capacity of developing-world healthcare systems, global health discourse has increasingly encompassed cancer care [1, 2]. In 2008, cervical cancer and childbirth mortality were comparable [3], and in 2012, the 5.3 million cancer deaths worldwide exceeded those caused by HIV/AIDS, tuberculosis, and malaria combined [1]. Yet, while low- and middle-income countries (LMICs) account for 80 % of disability-adjusted life-years lost to cancer, only 5 % of oncology resources are spent in those countries [2, 4]. Particularly in LMICs, cancer services are inaccessible for most patients, with existing programs located primarily in urban areas or the private sector and focusing on select cancers [4, 5]. Perhaps with the exception of the AMPATH-Oncology consortium in Kenya [6], models of oncology programs embedded within the public sector and serving rural poor patients are lacking. Furthermore, while general principles in cancer service delivery in resource-constrained settings have been described [4, 5, 7, 8], few groups outline program-level implementation of oncology services.

Rwanda has greatly improved the health of its 11 million citizens since the catastrophic 1994 genocide [9]. Yet cancer care was extremely limited as recently as 2012, with no oncologist, only one hematopathologist, and three clinical pathologists based in the country. At the time, services were available at only one district hospital and three urban-based national referral hospitals. In 2011 driven by its strong equity agenda and having initiated impressive cervical cancer prevention efforts [10], Rwanda’s Ministry of Health (RMOH) invited Partners In Health (PIH) and Dana-Farber/Brigham and Women’s Cancer Center (DFBWCC) to partner in expanding cancer care nationally, targeting poor, rural-based patients. In July 2012, the Butaro Cancer Center of Excellence (BCCOE), a public rural-based facility, was inaugurated by former US President Bill Clinton and the Honorable Minister of Health, Dr. Agnes Binagwaho. Here, we report program-level description of implementing BCCOE, its preliminary impact and challenges faced in order to share lessons and inform service delivery in similar settings.

## Methods: key components to delivering accessible cancer services in a resource-constrained setting

### Partnerships

Butaro Cancer Center of Excellence was founded on diverse, long-term partnerships [7, 8]. Spearheading the initiative, the RMOH set national priorities and coordinated collaborations. RMOH also provided infrastructure, staff (recruitment of nurses and junior doctors along with salary support for most of them) and non-specialized consumables complementary to oncology services (such as pain medications and intravenous fluids). PIH, an international non-governmental organization with a mandate to deliver healthcare to the most vulnerable communities and extensive experience working in resource-constrained settings [11], was initially invited by RMOH in 2005 to help expand HIV services to communities in a rural district in the Eastern province. This partnership grew over time to address evolving needs including those in primary care, medical education and specialty-related care such as cancer. In addition to bringing this implementation experience to cancer care, PIH brought on board a network of partners that availed technical expertise in oncology and pathology, funding to support salary for selected staff (such as Rwandan internist and pediatrician), procurement of specialized oncology medications and supplies, pathology equipment and reagents as well as relationships to defray costs (through procurement networks, volunteer clinicians). These partners, including Harvard Medical School, DFBWCC, Jeff Gordon Children’s Foundation, The Breast Cancer Research Foundation, LIVESTRONG, and GlaxoSmithKline, are diverse in scope and committed to long term partnerships.

### Setting and infrastructure

Butaro Cancer Center of Excellence is housed within Butaro hospital, a rural district hospital in Burera district (which is home to 321,000 people) located in northern Rwanda approximately 93 km (and approximately 2.5 h drive) from the capital city. The hospital was built as a joint venture between RMOH, PIH, and Clinton Health Access Initiative. Upon its inauguration in January 2011, the hospital had 152 beds and departments in emergency, general medicine, pediatrics, surgery, maternity, two operating theatres and a neonatal intensive care unit. As of June 2012, the hospital had 160 employees (including 67 medical and 30 paramedical). The state-of-the-art design and record for outstanding health achievements in Burera District [12] made Butaro hospital suitable for a model oncology program. A 27-bed cancer ward was converted for inpatient care, and a weekly cancer outpatient clinic was integrated into the non-communicable diseases clinic roster.

### Personnel and training

All doctors and nurses at BCCOE received foundational didactic training through the national baseline cancer training, a 5-day didactic program - developed by partners including RMOH, PIH, DFBWCC – that covers general principles in cancer epidemiology, diagnosis, treatment, and documentation. Selected nurses additionally underwent an 8-week practicum-based longitudinal chemotherapy mixing and administration course led by visiting DFBWCC oncology specialty nurses. These trainings have facilitated long-term capacity building so that as of December 2014, 270 clinicians have received national baseline cancer training, and 36 nurses received the longitudinal training. Furthermore, three BCCOE Rwandan nurses have been recognized as national expert trainers, one of whom co-leads BCCOE-based longitudinal training offered to nurses from the National Referral Hospital of Kigali (CHUK).

### Clinical services

Upon its opening, BCCOE provided histopathology-based diagnosis [13], X-ray and ultrasound imaging, chemotherapy, selected surgical procedures, palliative care and socioeconomic supports [14] delivered by a multidisciplinary team (Table 1). Patients requiring radiotherapy were referred to Mulago Hospital in Uganda. With no oncology specialists on-site, care was delivered through task-shifting and structured twinning, and long-term collaboration between BCCOE and DFBWCC [7, 8]. Generalist physicians prescribed chemotherapy and performed biopsies (including breast core-needle and bone marrow) while nurses mixed and administered chemotherapy. Clinicians followed standardized protocols and consulted teams of DFBWCC-based experts through weekly ‘tumor board-like’ conference calls and frequent emails.

**Table 1:** Staffing at BCCOE

		Initial projections^a^	Dec 2012	Dec 2013	Dec 2014
Clinical	Physicians				
	Internists	1	1	2	2
	Pediatricians	1	1	1	1
	General Practitioners	1	1	2	2
	General Surgeon^b^	0.5	0.5	0.5	0.5
	OB/GYN^b^	0.5	0.5		0.5
	Nurses				
	Inpatient	7	7	13	22
	Outpatient^c^	2	2	2	3
	Care Coordinator	1	1	1	1
	Histopathology Technicians	-	2	2	4
	Nutritionist	-	0	0	1
	Social Worker	-	0	0	1
Programmatic	Program Director	1	1	1	1
	Program Manager	1	1	1	1
	Administrative Assistant	-	0	1	1
	Research Assistant	-	0	1	1
	Data Officer	-	0	1	1

^a^Initial projections based upon MOH estimates for 27-bed unit and 25 % annual increase in patient population

^b^ provide care for cancer and non-cancer patients

^c^Outpatient nurses shared with the NCD clinic

### Treatment protocols

Care was standardized using protocols adapted to available resources and for non-oncologist clinicians [7, 8]. Led by RMOH and supported by BCCOE staff, protocols were drafted by international oncology experts and reviewed by the national Non-communicable Diseases (NCD) technical working group on an on-going basis. Given Rwanda’s current lack of a radiotherapy center, treatment maximized outcomes without radiotherapy. The methodology for development and the initial vetting of protocols occurred at a conference held in Kigali, attended by cancer experts from France, USA, South Africa, and Senegal. The first national cancer protocols were endorsed in June 2012 (Table 2).

**Table 2:** Outline of Rwanda national cancer protocols, using breast cancer as an example

Each protocol:	
• Places evidence-based practices in the context of national resources. Where clinical trials specific to resource-constrained settings have been conducted, associated protocols are applied *(*e.g. *nephroblastoma, acute lymphoblastic leukemia, and Burkitt lymphoma).*	
• Is organized in a consistent format, with each protocol including subsections on screening, presenting signs and symptoms, pathology-based diagnosis, staging, treatment, and long-term follow up.	
• Specifies the minimal essential work-up required to yield accurate, pathology-based diagnosis and inform management decision-making within the treatment options available. At BCCOE, testing for HER2 status is not routinely performed given limited availability of HER2-targeted therapies such as trastuzumab.	
• Reflects staging classification that is clinically relevant and in line with treatment options. Three broad classifications/treatment groups for breast cancer are: early, locally advanced and metastatic.	
• Takes into account the currently limited availability of radiotherapy. Mastectomy (with level I/II lymph node dissection) is prioritized as surgical treatment of choice over lumpectomy.	
• Allows flexibility to address socioeconomic and logistical challenges seen in these settings. Weekly dosing of paclitaxel is employed where possible, however every three weeks dosing is offered given fewer barriers associated with the fewer hospital visits.	

For more detailed reference, copies of individual protocols are available upon request.

### Socioeconomic supports and access

Complementing the medical services available, socioeconomic supports such as food packages and transport vouchers were critical for vulnerable patients (who were identified using standardized socioeconomic and clinical criteria). Additionally, given their prohibitive costs for the vast majority of patients, chemotherapy, pathology testing, and referral to Uganda for radiotherapy were free for all presenting patients and covered by funding from grants, foundations, and private donations. For all other hospital-related costs, most patients paid 10 %, with the remainder covered by the national community-based health insurance scheme, *Mutuelles de Sante (Mutuelles)*.

### Procurement

A formulary list was generated from standardized protocols. Most medications were off-patent and included in the World Health Organization’s essential medicines list. This list also included supplies such as infusion pumps and personal protective equipment. Procurement planning transitioned from ad hoc purchases before 2012 for the few cancer patients to stock orders made every 6–12 months by 2014. During the first year, consumption was tracked intensively with monthly manual stock counts and projections based on patient volume. These consumption data were reviewed quarterly, and orders made for anticipated stock outs within 6 months. Available drugs and consumables were procured through the public supply chain while PIH obtained the remainder using funding and DFBWCC donations. The above was performed by a PIH-employed pharmacist, working closely with and capacitating Butaro Hospital pharmacist, pharmacy technicians and relevant clinical program managers.

### Electronic Medical Records (EMR) system

An oncology-specific EMR system was built on an open-source OpenMRS platform, borrowing principles from HIV medical record systems [15, 16]. The database was devised to run off local servers, enabling work during internet interruptions. With the exception of chemotherapy ordering performed by clinicians, data entry of demographic data and clinical events was conducted by a dedicated data officer who had 2 years post-secondary school training. A team of a systems analyst, software developers, data officer, program managers, and clinicians developed and implemented this oncology-focused EMR system.

### Ethics

Data related to human subjects presented in this manuscript is covered under a study protocol approved by Institutional Review Boards in Rwanda (National Health Research Council and Rwanda National Ethics Committee) and USA (Partners Human Research Committee). Given the retrospective design of this study and the use of de-identified data for analysis, informed consent was not required by respective Institutional Review Boards.

## Results: early findings, challenges faced and lessons learned

### Impact

Between July 1, 2012 and June 30, 2014, 2326 patients presented to BCCOE for cancer-related evaluation or care. This is in contrast to 21 patients seen at Butaro hospital for cancer-related evaluation and care in the preceding 12 month period. Of these 2326 patients, 1640 (70.5 %) were female. Mean age was 43 years (standard deviation, SD, 19.8) and 270 (11.6 %) were children younger than 18 years of age (Table 3).

**Table 3:** Demographic and clinical characteristics of patients seen at BCCOE during first year

Characteristics	Patients presenting for cancer evaluation/care in year one (*n* = 1144)		Patients diagnosed with cancer in year one (*n* = 759)	
	n	%	n	%
Age	1144	100.00	759	100.00
<18	135	11.80	102	13.44
18–45	470	41.08	247	32.54
46–60	334	29.20	256	33.73
>60	205	17.92	154	20.29
Gender	1144	100.00	759	100.00
Male	355	31.03	240	31.62
Female	789	68.97	519	68.38
Residence Province	1144	100.01	759	100.00
Northern Province - within Burera District	168	14.69	67	8.83
Northern Province- OUTSIDE Burera District	226	19.76	136	17.92
Other Provinces within Rwanda	702	61.36	522	68.77
Outside Rwanda	13	1.14	11	1.45
Unknown or not documented	35	3.06	23	3.03
Type of referring facility	1144	100.00	759	100.00
Referral or District Hospital	735	64.25	549	72.33
Other type of facility (including outside Rwanda)	153	13.37	90	11.86
Not documented	256	22.38	120	15.81
Insurance Status	1144	100.00	750	98.82
Mutuelles	821	71.77	564	74.31
Other or not documented	323	28.23	186	24.51
HIV status at intake	1144	100.00	759	100.00
Positive	67	5.86	57	7.51
Negative	500	43.71	367	48.35
Unknown or not documented	577	50.43	335	44.14
Smoking history	1144	100.00	759	100.01
Current of previous	189	16.52	150	19.77
Never	645	56.38	471	62.06
Unknown or not documented	310	27.10	138	18.18
Performance status at intake (ECOG) ^a^			759	100.00
0			313	41.24
1			102	13.44
2			46	6.06
3			29	3.82
4			13	1.71
Unknown or not documented			256	33.73

^a^ECOG: Eastern Cooperation Oncology Group [17]

The total number of yearly outpatient visits at Butaro hospital increased from 17,895 in 2011 to 20,235 in the program’s first year. During this period, the proportion of cancer-related outpatient visits also rose from 0.5 to 16 %. The increase in cancer-related hospital admissions was even more pronounced with 41 % of 6583 admissions between July 1, 2013 and June 30, 2014 being cancer-related (Butaro Hospital Health Management Information System data, unpublished).

Of the 1144 patients who presented during BCCOE’s first year (July 1, 2012 to June 30, 2013), 759 (66.3 %) were diagnosed with cancer (Table 3). Of these, 519 (68.4 %) were female and 102 (13.3 %) children. Fifty-seven (7.5 %) were HIV-positive by self-report and 150 (19.8 %) had a smoking history. A high proportion (461, 60.7 %) presented with good functional status ECOG of ≤2 [17]. Five hundred and sixty-four patients (74.3 % of cancer patients, or 98.4 % of those with documented insurance status) were on *Mutuelles*. Sixty-seven patients (8.8 %) resided in Burera District and 11 (1.5 %) in neighboring countries. Five hundred and forty-nine (72.3 %) were referred from district and national referral hospitals.

Pathology documentation was available for 562 patients (49.1 % of all patients presenting during BCCOE’s first year, or 74.0 % of patients diagnosed with cancer). As of June 30, 2013, 95 (12.5 %) cancer patients had died. Cause of death was documented as cancer-related for 24 (25.6 %), and unknown for 66 (69.5 %). Thirty-six (37.9 %) patients died at home or in the community while 45 (47.4 %) died during admission at BCCOE or another facility.

## Discussion

### Patients served

Butaro Cancer Center of Excellence has begun to deliver cancer services to a large number of patients in need in Rwanda (Table 3). Patients come from across the country, most residing in rural districts and covered by *Mutuelle*s, thus indicating delivery to our target vulnerable populations. The unprecedented patient volume reflects the great need and highlights BCCOE’s service as a national referral hospital for cancer care.

### Cancers seen

Among adults, the most common diagnoses were breast cancer (189, 28.8 %), cervical cancer (141, 21.5 %), and non-Kaposi sarcoma skin cancer (46, 7.0 %). Among children, nephroblastoma (28, 27.5 %), acute lymphoblastic leukemia/ALL (25, 24.5 %), and Hodgkin lymphoma (10, 9.8 %) were the leading diagnoses (Table 4). Cancers seen at BCCOE reflect some of the regional trends, such as the two most common cancers being breast and cervical. In its first year, BCCOE would have seen half of all breast cancer cases expected to be diagnosed nationally based on GLOBOCAN’s estimates of 576 new breast cancer diagnoses annually in Rwanda [1], though the true national incidence and prevalence is currently unknown given robust registries to more accurately document cancer cases continue to be under development. The leading pediatric cancer at BCCOE was nephroblastoma. At 27 % of pediatric cancers, this proportion was comparable to sites in the region such as in Zambia [18], though significantly higher than 5 % among pediatric cancers in the United States [19]. The second most prominent pediatric cancer, ALL, was similarly common internationally [1, 19].

**Table 4:** Types of cancers diagnosed in patients enrolled at BCCOE during first year

Cancers only (*n* = 759)					
	n	%		n	%
Cancer Type Adults (18 years or older)	657	100.0	Cancer Type Children (<18 years)	102	100.0
Breast cancer	189	28.8	Nephroblastoma	28	27.5
Cervical Cancer	141	21.5	ALL^a^	25	24.5
Non-Kaposi skin cancer	46	7.0	Hodgkin lymphoma	10	9.8
Head and neck cancer	38	5.8	Rhabdomyosarcoma	7	6.9
Colorectal cancer	26	4.0	Burkitt Lymphoma	5	4.9
Other Gynecological Malignancies	26	4.0	Osteosarcoma	5	4.9
CML^a^	22	3.4	Head and neck cancer	4	3.9
Other Non Hodgkin’s lymphoma	21	3.2	Other Leukemias	4	3.9
Gastric cancer	21	3.2	Other Non Hodgkin’s lymphoma	3	2.9
Kaposi sarcoma	16	2.4	Other Bone Cancers	3	2.9
Prostate cancer	16	2.4	Kaposi’s sarcoma	2	2.0
Other GI^a^	14	2.1	Other Gynecological Malignancies	2	2.0
Hodgkin’s lymphoma	13	2.0	Breast cancer	1	1.0
Soft-tissue Sarcomas	12	1.8	CML^a^	1	1.0
Other GU^a^	11	1.7	Metastatic (unknown primary)	1	1.0
Other solid cancers	9	1.4	Skin cancer	1	1.0
Other Leukemias	8	1.2			
ALL^a^	7	1.1			
Metastatic (unknown primary)	5	0.8			
Multiple myeloma	5	0.8			
Osteosarcoma	3	0.5			
Unknown type	2	0.3			
Rhabdomyosarcoma	2	0.3			
Other Bone Cancers	2	0.3			
Large B-cell lymphoma	1	0.2			
Nephroblastoma	1	0.2			

^a^ *CML* chronic myeloid leukemia, *Other GI* other gastrointestinal cancers, *Other GU* other genitourinary cancers, *ALL* acute lymphoblastic leukemia

The distribution of cancers seen at BCCOE was influenced by variation in clinical resources across facilities in Rwanda, as well as patient selection. Prostate and gastric cancers, among the top five cancers in the region [1] were anecdotally less commonly seen at BCCOE than the national referral hospital CHUK, which has resident endoscopists and urologists. Strengthening of the national cancer registry will provide a more accurate epidemiologic picture of cancers in Rwanda, though diagnostic capacity is limited in much of the country so many patients remain undiagnosed and therefore uncounted. In addition to teasing out the role of diagnostic and referral bias, further studies may be needed to explore region-specific risk factors for cancers.

### Outcomes

It is too early to describe disease-specific clinical outcomes, planned for the near future. Of note, however, of the 95 documented deaths, that the majority (53, 55.8 %) of patients die at home or while admitted at another facility makes discerning cause of death difficult and partly explains the large number (66, 69.5 %) of deaths with unknown cause.

### Challenges and lessons learned

#### Documentation gaps

The current oncology EMR needs further development but serves as a starting point. Many EMR systems for HIV in resource-constrained settings have demonstrated positive impact [15, 16], however few if any published examples describe systems for cancer care. While data gaps have to be addressed and clinical impact of BCCOE’s EMR system to be assessed, we have used this system to generate the presented data and routinely to support clinical management and program development. EMR data has, for instance, improved tracking of patients who miss appointments and prioritization of protocol revisions. While the initial phase of EMR development emphasized data supporting management decisions and patient retention, the next phase aims to better capture disease-specific outcomes, evaluate protocol adherence, and monitor treatment toxicity.

#### Staff shortages

Unprecedented patient volume contributed to perpetual staffing shortages, requiring periodic review. The initial seven ward nurses increased to 26 as of August 2014, corresponding to a nurse: inpatient ratio of 1:8. Despite the higher patient volume, in-service training, and heavy emotional toll of their work, oncology nurses are currently paid the same as those in other departments. Temporary relief has been achieved by increasing staff using personnel allocated by RMOH and additional funding from partners. However, strategies for compensation and professional accreditation for nurses and doctors will be necessary to sustain a cadre of oncology-skilled clinicians [8].

Butaro Cancer Center of Excellence does not have an on-site gynecologist or pathologist, and has only intermittently had a surgeon [13, 14]. While recruitment is in process and longer-term local capacity is developed through in-country post-graduate programs [8], discussions are underway for BCCOE to serve as a national oncology rotation site for post-graduate doctors. The stream of students and affiliated rotating faculty may mitigate staffing shortages while presenting valuable opportunities for learning and collaboration.

#### Access to radiotherapy

Over 50 % of cancer cases in LMICs countries are estimated to require radiotherapy [20], yet Rwanda does not have a radiotherapy facility. PIH sponsored an average of 15 patients a month to receive radiotherapy at Uganda’s Mulago Hospital. Due to budget constraints, a committee of clinicians used institutional guidelines to select a subset of eligible patients, mainly with curable cervical and head and neck cancers. The average cost per patient receiving a 6-week course of chemo-radiation for locally advanced cervical cancer (includes transport, room and board, and medical services) was USD 2800, amounting to over USD 500,000 spent per year. Systematic study of patient outcomes following referral for radiation at Mulago is planned. Discussions are ongoing to build a radiotherapy facility within the next 5 years— a critically-needed investment to expand treatment options for Rwandans.

#### Program expenses

The most significant costs in BCCOE’s program budget were radiotherapy referrals, chemotherapy, and staff salaries. Chemotherapy orders during BCCOE’s first year amounted to USD 110,000 while clinical staff salaries totaled USD 312,000. Though a substantial amount, funding can be within reach and costs significantly reduced through partnerships. As examples, salaries for US-licensed specialists supporting cancer care were subsidized through part-time hospitalist work in the US. Medications were sourced from accredited India-based generic drug companies whose prices were three to five times cheaper than European counterparts [21]. In addition to these program-level strategies, it is our hope that through advocacy and price negotiation, global financing mechanisms for cancer care will become a reality, as was accomplished for antiretroviral therapy.

#### Sustainability

The scale of BCCOE’s work and its funding can be attributed to the broad and invested collaborations supporting it. While we currently have not yet enumerated the cost of implementing BCCOE (an exercise that is currently in process), approaches have been made to minimize costs and facilitate sustainability of the program. RMOH’s engagement and role in financing and shepherding partnerships has not only helped frame national priority, but has provided an avenue for continuation of services over the long-term and their integration into the existing health infrastructure. Use of open-source data systems and of rotating volunteer experts have also minimized costs. On a global scale partners are participating in efforts with World Health Organization and manufacturing companies geared toward lowering prices of chemotherapeutic agents and improving access internationally.

## Conclusion

Butaro Cancer Center of Excellence demonstrates that, with partnerships and supports, complex cancer care can be delivered in the most resource-constrained settings and despite significant challenges. Key attributes that have made BCCOE possible were: a) meaningful partnerships emphasizing health systems strengthening, b) innovative task- and infrastructure-shifting, c) strong RMOH leadership coordinating efforts to embed services within the public sector, and d) an equity-driven agenda to service those most in need. This combination is rare yet essential to expand desperately needed cancer services globally. Looking forward, clinical outcomes will be assessed such as long-term survival, retention and treatment-related toxicity for specific cancers treated. The developed EMR system will serve as a valuable platform for this assessment. Finally, these partnerships continue to grow and support national efforts, including periodic review of national protocols to reflect experience since 2012 and planning for an in-country radiotherapy center.

### Availability of data and materials

Presented data are available upon request from corresponding author.

## Abbreviations

LMICs: low- and middle-income countries
RMOH: Rwanda Ministry of Health
PIH: partners in health
DFBWCC: Dana-Farber/Brigham and Women’s Cancer Center
BCCOE: Butaro Cancer Center of Excellence
EMR: electronic medical records
NCD: non-communicable diseases
SD: standard deviation
ALL: acute lymphoblastic leukemia
